# Grip Strength Is Associated With Cognitive Performance in Schizophrenia and the General Population: A UK Biobank Study of 476559 Participants

**DOI:** 10.1093/schbul/sby034

**Published:** 2018-04-19

**Authors:** Joseph Firth, Brendon Stubbs, Davy Vancampfort, Josh A Firth, Matthew Large, Simon Rosenbaum, Mats Hallgren, Philip B Ward, Jerome Sarris, Alison R Yung

**Affiliations:** 1 NICM Health Research Institute, School of Science and Health, University of Western Sydney, Sydney, Australia; 2 Division of Psychology and Mental Health, Faculty of Biology, Medicine and Health, University of Manchester, Manchester, UK; 3 Physiotherapy Department, South London and Maudsley NHS Foundation Trust, London, UK; 4 Department of Psychological Medicine, Institute of Psychiatry, Psychology and Neuroscience, King’s College London, London, UK; 5 KU Leuven Department of Rehabilitation Sciences, Leuven, Belgium; 6 UPC KU Leuven, Kortenberg, Belgium; 7 Department of Zoology, Edward Grey Institute, University of Oxford, Oxford, UK; 8 Merton College, University of Oxford, Oxford, UK; 9 The Prince of Wales Hospitals, Randwick, Australia; 10 School of Psychiatry, University of New South Wales, Sydney, Australia; 11 Black Dog Institute, Randwick, Australia; 12 Department of Public Health Sciences, Karolinksa Institute, Stockholm, Sweden; 13 Schizophrenia Research Unit, Ingham Institute of Applied Medical Research, Liverpool, Australia; 14 Department of Psychiatry, University of Melbourne, The Melbourne Clinic, Melbourne, Australia; 15 Greater Manchester Mental Health Foundation Trust, Manchester, UK

**Keywords:** psychotic disorders, psychosis, neuropsychological, resistance training, exercise, cognitive decline

## Abstract

**Background:**

Handgrip strength may provide an easily-administered marker of cognitive functional status. However, further population-scale research examining relationships between grip strength and cognitive performance across multiple domains is needed. Additionally, relationships between grip strength and cognitive functioning in people with schizophrenia, who frequently experience cognitive deficits, has yet to be explored.

**Methods:**

Baseline data from the UK Biobank (2007–2010) was analyzed; including 475397 individuals from the general population, and 1162 individuals with schizophrenia. Linear mixed models and generalized linear mixed models were used to assess the relationship between grip strength and 5 cognitive domains (visual memory, reaction time, reasoning, prospective memory, and number memory), controlling for age, gender, bodyweight, education, and geographical region.

**Results:**

In the general population, maximal grip strength was positively and significantly related to visual memory (coefficient [coeff] = −0.1601, standard error [SE] = 0.003), reaction time (coeff = −0.0346, SE = 0.0004), reasoning (coeff = 0.2304, SE = 0.0079), number memory (coeff = 0.1616, SE = 0.0092), and prospective memory (coeff = 0.3486, SE = 0.0092: all *P* < .001). In the schizophrenia sample, grip strength was strongly related to visual memory (coeff = −0.155, SE = 0.042, *P* < .001) and reaction time (coeff = −0.049, SE = 0.009, *P* < .001), while prospective memory approached statistical significance (coeff = 0.233, SE = 0.132, *P* = .078), and no statistically significant association was found with number memory and reasoning (*P* > .1).

**Conclusions:**

Grip strength is significantly associated with cognitive functioning in the general population and individuals with schizophrenia, particularly for working memory and processing speed. Future research should establish directionality, examine if grip strength also predicts functional and physical health outcomes in schizophrenia, and determine whether interventions which improve muscular strength impact on cognitive and real-world functioning.

## Introduction

Maximal isometric handgrip provides a simple and valid proxy of muscular strength, and is recognized as the most suitable measure of muscular function for clinical settings.^1^ This brief and easily-administered assessment is becoming a commonly used outcome measure for multiple health domains in the general population.^2^ For instance, large cohort studies have shown that weak grip strength is associated with poor health-related quality of life,^3^ and can be used to identify individuals at risk of mobility limitations, fracture, frailty, and falls risk.^4,5^ Furthermore, handgrip strength is emerging as a useful clinical marker of mortality risk,^2,6^ as grip strength can be an even stronger predictor than either systolic blood pressure^2^ or obesity^6^ for both all-cause and cardiovascular mortality. Thus, there is a growing interest in how this brief, functional task can capture several important domains.

However, the research to date has only established these relationships in older adults, and the generalizability to other populations is unknown. In particular, there is an absence of research examining the relationships between muscular strength and health outcomes in psychiatric populations. Nonetheless, research in the general population has shown that association between muscular strength and health extends beyond physical functioning, to also include brain health. For instance, elderly adults with greater muscular strength in the upper and lower limbs have increased cognitive capacities and are at lower risk of developing age-related cognitive impairment.^7,8^ Importantly, the relationship persists even when controlling for other putative factors such as age, adiposity, and physical activity..^7,8^ Indeed, handgrip strength is positively correlated with performance across multiple cognitive domains in aging samples, including verbal and spatial abilities, processing speed, and memory.^9,10^ Indeed, a recent systematic review of 15 longitudinal studies found that the consistent predictive relationships between grip strength and cognition indicated that decrements in grip strength can provide a clinically useful marker of overall cognitive decline.^11^

Despite the consistency and strength of the associations observed between grip strength and cognition in older adults, the neurophysiological underpinnings of this relationship are currently unclear. Although greater total brain volume is associated with larger muscle size, the relationships between total brain volume and muscular strength are inconsistent.^12^ Nonetheless, studies have linked muscular strength with white matter regions of the brain,^12^ particularly in showing that individuals with greater grip strength have significantly fewer signs of aging-related white matter degradation, identified by “white matter hyperintensities” (WMH).^10,13^ As WMHs can impede performance across a broad spectrum of both physical and mental tasks,^14^ this may explain part of the relationship between grip and cognition.

However, recent studies have shown that the correlation between decreases in handgrip and cognition which consistently occur overtime cannot be accounted for white matter degradation alone.^10^ Another mechanism through which grip may relate to cognition in older adults is through a shared link to inflammation, as both aging-related cognitive decline and weakened grip are predicted by heightened levels of inflammatory markers, such as interleukin-6.^15,16^

Although there is a growing body of literature in relation to handgrip strength and cognition in aging populations, no studies have yet examined the associations between handgrip strength and cognition among individuals with schizophrenia. Schizophrenia is associated cognitive deficits across a broad range of neurocognitive domains, including processing speed, short and long memory, and reasoning.^17,18^ These deficits predate the onset of illness and persist over time despite antipsychotic treatment.^19,20^ They are highly predictive of the long-term functional disability often observed in people with schizophrenia,^21^ and may impede psychosocial recovery to an even greater extent than positive psychotic symptoms.^22,23^ However, it remains unclear if the cognitive deficits observed in schizophrenia are associated with reduced muscular strength.

Therefore, this study aims to use population-scale data from the United Kingdom (UK) Biobank to examine the relationship between maximal grip strength with cognition in people with schizophrenia and those without; thus determining if the relationships which exist between muscular strength and cognitive function in the general population also apply to individuals with schizophrenia. We further aim to establish which specific domains of neurocognitive performance, including processing speed, memory, and executive function, are related to grip strength in both individuals with schizophrenia and the general population.

## Methods

We performed a cross-sectional analysis of data collected between 2007 and 2010 from the baseline assessments for the UK Biobank. This particular study was approved by the UK Biobank research committee, and is covered under the generic ethical approval from the NHS Research Ethics Committee (Ref. 11/NW/0382). The UK Biobank is a nationwide, health-orientated, cohort study aiming to investigate how peoples’ lifestyles, environment and genetics are related to various health outcomes. Invitations were mailed out to over 9.2 million homes throughout the UK; recruiting 502664 adults, aged 37–73 across 22 dedicated assessment centers throughout the UK. Upon visiting the assessment centers, participants gave informed consent before completing extensive testing, including a computerized (touchscreen) questionnaire, face-to-face interviews with research staff, and physical health assessments. The full details of study design and data collection processes for the UK Biobank are detailed elsewhere.^24^

### Participant Categorization

First, participants with neurological conditions known to impair cognitive performance were excluded from both the schizophrenia sample and control sample groups. The neurological conditions and their respective biobank variable codes are listed in supplementary table 1. The UK Biobank is integrated with hospital records to enable access to all recorded clinical diagnoses. In this study, we categorized our schizophrenia sample as individuals from the UK Biobank with a recorded ICD-10 diagnosis (primary or secondary) of any nonaffective psychotic disorder, including schizophrenia and schizophrenia-like conditions (ICD-10 disease classes F20-F29). The “general population” sample comprised of all remaining UK Biobank participants without a recorded history of nonaffective psychotic disorders.

### Handgrip Strength Measurement

Maximal handgrip strength measurement was performed at UK Biobank assessment centers by research assistants using the “Jamar J00105 hydraulic hand dynamometer.” Assessments followed the standard procedures for obtaining maximal grip scores which have been shown to have high reliability/reproducibility.^1^ Each dynamometer was calibrated at the beginning of every testing day. The measurement process commenced by allowing participants to select their preferred grip position of the 5 positions available (ranging between 1.375 and 3.375 inches). A maximal score from both left and right hands was then obtained while participants were seated in an upright position, with elbow positioned laterally adjacent to the torso, and their forearm placed on an armrest. A single trial was conducted for each hand; which obtains an equally reliable and valid measurement of maximal grip strength as repeated-testing protocols.^25^ Participants were asked (using a single question) where they regarded themselves as left or right-handed. The maximal score from their dominant hand was used in all analyses. Where participants failed to specify a dominant hand, or described themselves as ambidextrous, their highest score across both hands was selected and used in analyses.

### Cognitive Functioning

Cognitive functioning was assessed using a 15-min computerized battery which was developed specifically for the UK Biobank study to enable population-scale cognitive testing that could be administered without researcher supervision. Full details are available elsewhere.^26^ Briefly, the battery consisted of 5 individual tasks, quantifying performance in 5 separate cognitive domains as follows:

1. *Reaction time*; mean response time in milliseconds to a prespecified visual stimuli (sequentially matching cards).
2. *Reasoning*; number of verbal and numerical logic problems solved in 2 min.
3. *Numeric memory*; maximum length digit string correctly recalled.
4. *Visuospatial memory*; number of attempts required to correctly match 6 pairs of symbol cards following brief visual presentation of these stimuli.
5. *Prospective memory*; dichotomous assessment measuring if participants succeeded or failed to act on an earlier instruction after a delay period.

### Confounding Variables

Gender, age, bodyweight, education, and geographical location were identified as potentially confounding variables. Information on age and gender were collected using a computerized questionnaire, completed at UK Biobank assessment centers. Highest qualifications/education was also collected from this questionnaire. Responses were dichotomized to categorize as those with and those without university or college degree-level qualifications. Bodyweight was measured by a research assistant during physical health assessments. Geographical region was assessed as “Biobank assessment center,” as per the data collection process.

### Statistical Analysis

Analyses were conducted in R (version 3.1.2). We aimed to assess how grip strength related to each of the 5 cognitive domains. We used the same analytical framework across these cognitive measures, but carried out separate models for each. Reasoning, number memory, and reaction time were assessed as the response variable in linear mixed models (LMMs). Reaction time was log transformed to normalize data. Visuospatial memory was set as the response variable in a generalized linear mixed model (GLMMs) with Poisson error structure (due to this being count data) and GLMMs with binomial error structure and logit link function were used for prospective memory (due to the binomial nature of this particular outcome).

Across all models, we aimed to examine the relationship between the response variables (ie, each cognitive measure) and maximal grip strength while simultaneously accounting for variables (for which sufficient data was available) which may affect this relationship. Thus, we fitted grip strength as a fixed effect within the models, and also included gender, age, bodyweight, and educational status as additional predictors. To account for the expected nonindependence between different testing sites (as well as other variables associated with this), Biobank testing centre was also fitted as a random effect. We report the relevant summary statistics for each analysis (ie, the averages and error surrounding measurements of interest and the composition and size of the sample) along with the model parameters of interest and the associated confidence of these estimates.

## Results

### Included Participants

The final analyses included a maximum of 476559 participants who fit eligibility criteria and had completed both the grip strength measurement and at least one cognitive task. The most commonly completed task was visual memory (table 2), whereas only around 10% of participants (*n* = 48444) completed the number memory task, as this was administered only during the UK Biobank initial pilot data collection phase. The clinical sample consisted of 1162 participants with ICD-10 hospital diagnoses of nonaffective psychotic disorders. The mean age at assessment was 54.3 years (95% range = 41–69 years) and 54.3% were male. The control sample consisted of 475397 participants with no history of psychotic disorders. Among these, the mean age was 56.5 years (95% range = 41–69 years) and 45.4% were male. Table 1 displays the sociodemographic characteristics of schizophrenia and control samples.

**Table 1.** Sample Characteristics

	Schizophrenia Sample (*n* = 1162)			General Population (*n* = 475397)		
	Raw Mean	Lower Quantile	Upper Quantile	Raw Mean	Lower Quantile	Upper Quantile
Age (years)	54.3	41	69	56.49	41	69
Gender	54.3% male	—	—	45.4% male	—	—
Bodyweight (male), kg	87.0	59.2	126.4	78.3	62.7	119.0
Bodyweight (female), kg	75.6	49.0	114.7	71.4	50.7	105.8
Grip strength (male), kg	36.1	14	56	40.7	22	60
Grip strength (female), kg	22.2	8	36	24.5	11	38

*Note:* kg, kilograms. Upper and lower quantiles set at 2.5%, representing 95% range.

**Table 2.** Linear Mixed Models Analyses; Associations Between Grip Strength and Each Cognitive Domain in Schizophrenia and General Population

General Population					Schizophrenia Sample			
Cognitive Task	Total *n*	Coeff. (SE)	*T*-Value	*P*-Value	Total *n*	Coeff. (SE)	*T*-Value	*P*-Value
Visual memory^a^	475397	**−0.1601 (.003**)	−52.6078	<.001	1,162	**−0.155 (0.042**)	−3.6758	<.001
Reaction time^a^	471251	**−0.0346 (.0004**)	−87.773	<.001	1,113	**−0.049 (0.009**)	−5.4758	<.001
Prospective memory	163594	**0.3486 (.0092**)	38.0492	<.001	423	0.233 (0.132)	1.7623	.078
Reasoning	158438	**0.2304 (.0079**)	29.3348	<.001	363	0.159 (0.140)	1.1381	.256
Number memory	48347	**0.1616 (.0092**)	17.6486	<.001	97	0.087 (0.237)	0.3678	.714

*Note:* Coeff., coefficient from linear mixed model; *N*, number of participants; SE, standard error. Bold indicates statistically significant

^a^Negative association as lower scores = better cognitive performance.

### Relationships Between Grip Strength and Cognitive Functioning

**Fig. 1.**
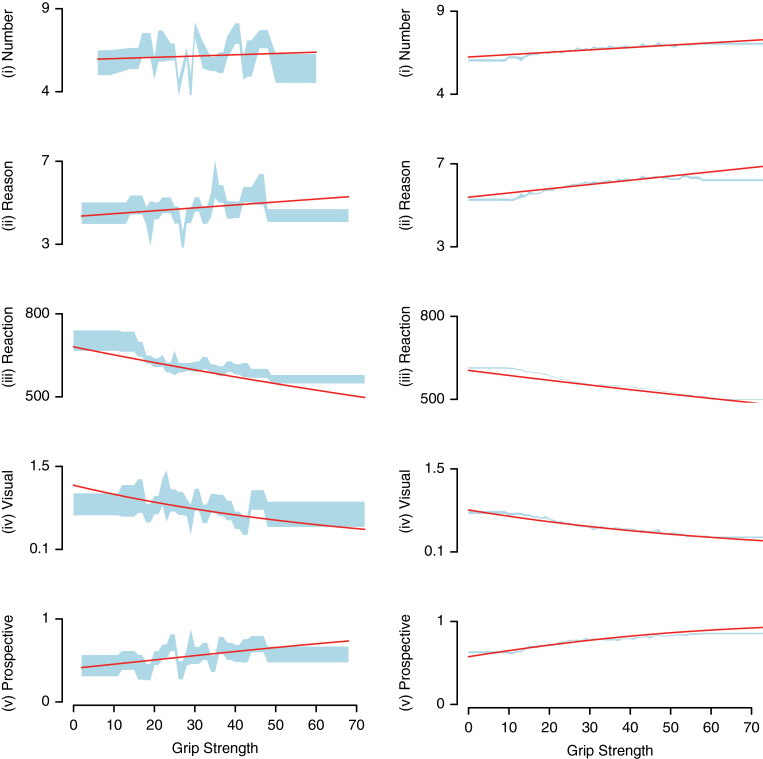
The relationship between grip strength and cognitive domains in (a) schizophrenia sample and (b) general population. *Y*-axis denotes the cognitive measure considered. Red lines show the fit of the linear mixed models (i–iii) and generalized linear mixed models (iv and v) (see table 2 for full model details). Blue shaded area shows the standard error around the mean of the raw cognitive measure split into bins of 15 data points per section.

Table 2 details full results of the (G)LMMs examining relationships between grip strength and cognition in the schizophrenia sample and general population. As shown in figures 1a and 1b, all relationships were in the hypothesized direction, with greater grip strength being associated with better cognitive performance.

Considering individuals from the general population, the (G)LMMs (with gender, age, weight, education as fixed effects, and geographical region as a random effect) showed that higher grip strength was significantly and positively related with better task performance for visual memory (*t* = −52.61, coefficient [coeff] = −0.1601, standard error [SE] = 0.003), reaction time (*t* = −87.8, coeff = −0.0346, SE = 0.0004), reasoning (*t* = 29.33, coeff = .2304, SE = .0079), number memory (*t* = 17.65, coeff = .1616, SE = 0.0092), and prospective memory (*t* = 38.05, coeff = 0.3486, SE = 0.0092: all *P* < .001).

Among individuals with schizophrenia, we found significant relationships between grip strength and both cognitive tests completed by the majority of patients (*n* ≥ 1100). Maximal grip was a highly significant predictor of visual memory (*n* = 1162; *t* = −3.7, coeff = −0.155, SE = 0.042, *P* < .001) and reaction time (*t* = −5.5, coeff = −0.049, SE = 0.009, *P* < .001). As shown in figure 1b, relationships between grip and cognition for the other cognitive tasks that were completed by fewer participants were all in the predicted direction. Prospective memory approached statistical significance (*t* = 1.76, *n* = 423, coeff = 0.233, SE = 0.132, *P* = .078), while no statistically significant associations were observed for reasoning (*n* = 363, *t* = 1.14, coeff = 0.159, SE = 0.140, *P* = .256) or number memory (*n* = 97, *t* = 0.37, coeff = 0.087, SE = 0.237, *P* = .714), potentially attributable to reduced statistical power due to the lower number of participants with schizophrenia completing these tasks. We sought to assess if those relationships between grip strength and cognition that were nonsignificant in the schizophrenia group were quantifiably weaker than the control sample. Thus, we conducted supplementary analyses considering all individuals within the same model and adding “condition” (ie, schizophrenia or nonschizophrenia) as an interaction term. These analyses found no interaction between grip strength and schizophrenia for performance in prospective memory (*P* = .506), number memory (*P* = .569) or reasoning (*P* = .357), ie, we found no evidence the relationships between grip and cognition in the schizophrenia sample differed significantly from those observed in the general population.

Due to the older average age of the UK Biobank sample, we conducted post hoc sensitivity analyses examining if the relationships between grip and cognition persisted when restricting the analyses entirely to the younger half of the sample, ie, those aged ≤55 years. Handgrip still held strong and statistically significant relationships with all 5 domains of cognitive functioning (all *P* < .001) among individuals aged ≤55 years (see supplementary table 2 for details).

Further post hoc sensitivity analyses were conducted to explore if the significant associations observed between handgrip strength and cognitive performance persisted even when controlling for cardiometabolic health, by adding the following additional confounding factors to our original models: (1) waist circumference as a proxy of metabolic risk,^27^ and (2) any previous diagnostic record of high blood pressure, angina, stroke, or heart attack. Controlling for these additional factors did not affect results (see supplementary table 3 for full details).

## Discussion

To our knowledge, this is the first study examining grip strength as a predictor of cognitive functioning in people with schizophrenia. Furthermore, the expansive sample of over 450000 controls makes this by far the largest study examining relationships between muscular strength and cognition in any population to date. Confirming the findings of previous research,^11^ we found consistently positive relationships between higher grip strength and better cognition in the general population. There were significant associations observed across all 5 cognitive tasks, even when accounting for age, gender, educational, and anthropomorphic differences. Furthermore, our subgroup analyses showed that significant associations between grip strength and cognition existed even in the younger half of the sample (ie, those aged 40–55 years), making this among the largest studies to date demonstrate a connection between muscular and cognitive functioning in middle-aged adults, as the majority of research in this area has been conducting in aging samples.^11^ The strength of these findings is supported by the rigorous methodology applied by the UK Biobank^24^ and population-scale data set.

One limitation to the findings is the relationships between grip strength and cognitive functioning fell short of statistical significance in the schizophrenia sample for the tasks which were completed by fewer participants. Nonetheless, we did find significant relationships in the tasks with the largest samples, with data from over 1100 individuals with schizophrenia showing strong associations between handgrip strength and performance in the pairs matching task (which measures working memory) and reaction time task (reflecting processing speed). This is of interest, as impairments in both working memory and processing speed are pervasive among people with schizophrenia,^28^ and these domains are among the most important neurocognitive predictors of real-world social and occupational functioning in this population.^29–31^

However, when interpreting these task-by-task differences, it must be considered that individuals’ performance across the different tasks in the brief cognitive battery used by the UK Biobank is to some extent underpinned by overall cognitive ability.^26^ Similarly, the cognitive deficits observed in schizophrenia can to some extent be understood as a generalized cognitive deficit across multiple domains.^32^ Thus, rather than providing evidence for grip strength relating to specific aspects of cognition, the lack of statistical significance for some cognitive domains in the schizophrenia sample may instead be attributable to differences in amounts of available data for each cognitive task (table 1). Indeed, the schizophrenia sample consistently showed trends in the same direction as the healthy controls for grip strength and each cognitive domain. Additionally, analyses assessing whether these cognitive measures were influenced by the potential interaction between grip strength and whether or not these individuals had schizophrenia found no evidence for differential effects of grip strength in schizophrenia vs nonschizophrenia samples. This generalized association between grip and cognition is also consistent with the relationships observed between grip and multiple other broad clinical outcomes, including functional disability and overall well-being. Beyond establishing if the link between grip and cognition is specific to any individual domain, it is also important for future research to explore if any cognitive domains are particularly sensitive to resistance training interventions, to determine how such interventions could be useful for people with schizophrenia.

Furthermore, as handgrip strength has also been shown to be a valid indicator of overall physical health status and mortality risk,^2,6,33^ future studies should evaluate if grip strength relates to physical health in schizophrenia. The life-expectancy gap for people with schizophrenia is currently around 15–25 years younger than the general population, largely due to physical health disorders.^34,35^ However, the key determinants of physical health outcomes in schizophrenia are not fully established, partly because current self-report measures of health behaviors are largely invalid in this population.^36^ Thus, if muscular strength (measured as handgrip) was found to also predict overall physical health status and mortality risk, this would add further value to the clinical utility of this measure.

Given the potential value of handgrip strength as a potential marker of cognitive dysfunction, the causal directionality and mechanisms underpinning these relationships should be investigated. The relationship between muscular strength and cognitive functioning in the general population is bolstered by increasing evidence for strength training interventions improving cognitive functioning. For instance, RCTs in aging populations showing significant improvements in cognitive functioning from resistance exercise, suggesting that targeting muscular strength can be an effective method for treating cognitive deficits.^37,38^ More recently, Suo et al. found that engaging older people (*n* = 100) in resistance training was even more effective for reducing cognitive impairments than targeting cognition directly through “computerized brain training.” Furthermore, the cognitive improvements in the resistance training group were mediated by reversal of aging-related white matter degradation (identified by white matter hyperintensities) in various areas across the brain.^39^ As these changes were not observed in the cognitive training group, the findings present a clear mechanistic pathway through which muscular strength training could improve cognitive capacities in aging samples. Importantly, the mechanisms indicated by this experimental study are also consistent with previous observational studies, showing that higher muscular function (as measured by grip strength) is associated with fewer white matter hyperintensities.^10,13^

Further research is now required to establish if the same neurobiological pathways which underlie the connection between grip strength and cognition in healthy samples also applies to individuals with schizophrenia, as new interventions for cognitive enhancement in schizophrenia are needed in this population. While pharmacological approaches to treating cognitive deficits in schizophrenia have limited efficacy,^40^ RCTs of aerobic exercise interventions in schizophrenia have found improved performance across multiple cognitive domains.^41,42^ Currently, there is an absence of RCTs examining cognitive outcomes of strength training in schizophrenia.^43^ Nonetheless, single-arm pilot studies have reported cognitive improvements following strength training in schizophrenia samples.^44^ Controlled studies combining resistance training components within aerobic exercise sessions have also suggested beneficial effects on neurocognition and neural connectivity.^45–47^

Another limitation to our data was the lack of information on schizophrenia-specific factors that could potentially impact upon the link between grip strength and cognition. For instance, negative symptoms and extrapyramidal symptoms could feasibly impede performance in both grip strength and cognitive tasks for some patients with schizophrenia. A further limitation to our data set was the middle- to older-age range of the UK Biobank, meaning that the relationships between muscular and cognitive functioning in younger people with schizophrenia could not be assessed. However, population-scale studies samples have previously indicated that muscular strength is related to mental health from even a young age, as greater grip strength in adolescence reduces the risk of developing adulthood psychiatric conditions (including both psychotic and mood disorders) by 15%–65%.^33^ Thus, future research also could investigate the potential benefits strength training interventions in the early stages of psychotic illnesses, as this time period may represent a critical period of increased neuroplasticity and greater potential for cognitive enhancement.^48,49^

A final limitation is the lack of data on “real world functioning” of the participants. Cognitive functioning is highly predictive of functional outcomes in people with schizophrenia,^21^ and previous studies of aerobic fitness in schizophrenia have shown significant correlations with both cognitive and real-world functioning.^50^ Therefore, future studies should now investigate if and how muscular strength may also relate to social and occupational functioning in people with schizophrenia—perhaps even as a tool for predicting functional decline, as has been found in other populations.^10,51^

In conclusion, this population-scale study confirms, in a large sample, the significant correlation between grip strength and multiple cognitive domains in the general population. Further research is required to understand the causal direction of these relationships, as well as determining the implications of grip strength for real world functioning in psychiatric populations. Additionally, as there are no RCTs examining cognitive outcomes of strength training in schizophrenia, future studies should investigate if muscle-strengthening interventions can also confer cognitive enhancing effects, particularly in the early stages of illness. Such trials will help to determine the directionality of the relationship between muscular strength and cognitive functioning, and potentially offer a novel method for reducing cognitive impairments.

## Supplementary Material

Supplementary data are available at *Schizophrenia Bulletin* online.

Supplementary Table 1

Supplementary Table 2

Supplementary Table 3

## Funding

J.F. is supported by a Blackmores Institute Fellowship and an MRC Doctoral Training Grant (P117413F07). J.S. is funded by an NHMRC Research Fellowship (APP1125000). B.S. is supported by the National Institute for Health Research (NIHR) Collaboration for Leadership in Applied Health Research and Care South London at King’s College Hospital NHS Foundation Trust. B.S. is also part funded by the National Institute for Health Research (NIHR) Biomedical Research Centre at South London and Maudsley NHS Foundation Trust and King’s College London. S.R. is funded by an NHMRC Early Career Fellowship (APP1123336).
