# Supplementary material for: Grip Strength Is Associated With Cognitive Performance in Schizophrenia and the General Population: A UK Biobank Study of 476559 Participants
Source: Schizophr Bull. 2018 Apr 19;44(4):728–36. doi: 10.1093/schbul/sby034 (PMC6007683; doi:10.1093/schbul/sby034)
Supplement: Supplementary Table 2 [file sby034_suppl_supplementary-table-2.doc]

| **Supplementary Table 2.** Associations between grip strength and cognition in those aged 55 years and under | | | | | | |  | | |
| --- | --- | --- | --- | --- | --- | --- | --- | --- | --- |
| Cognitive task | Coeff. | S.E. | T value | p-value |  |  | |  |  |
| Visual memory* | -0.209 | 0.0050 | -41.77 | <0.001 |  |  | |  |  |
| Reaction time* | -0.044 | 0.0001 | -77.24 | <0.001 |  |  | |  |  |
| Prospective memory | 0.378 | 0.0147 | 25.79 | <0.001 |  |  | |  |  |
| Reasoning | 0.216 | 0.0121 | 17.75 | <0.001 |  |  | |  |  |
| Number memory | 0.157 | 0.0138 | 11.37 | <0.001 |  |  | |  |  |
| Notes: Coeff, Coefficient from Linear Mixed Model; S.E. standard error   *Negative association as lower scores = better cognitive performance; **BOLD** indicates statistically significant | | | | | | |  | | |
