# Supplementary material for: Grip Strength Is Associated With Cognitive Performance in Schizophrenia and the General Population: A UK Biobank Study of 476559 Participants
Source: Schizophr Bull. 2018 Apr 19;44(4):728–36. doi: 10.1093/schbul/sby034 (PMC6007683; doi:10.1093/schbul/sby034)
Supplement: Supplementary Table 3 [file sby034_suppl_supplementary-table-3.doc]

| **Supplement 3.** Results of sensitivity analyses, showing relationships between grip strength and cognitive domains in fully adjusted models^1^ | | | | | | | | |
| --- | --- | --- | --- | --- | --- | --- | --- | --- |
| General population | | | |  | Schizophrenia sample | | | |
| Cognitive task | Coeff. | S.E. | T value | p-value | Coeff. | S.E. | T value | p-value |
| Visual memory* | -0.135 | 0.0031 | -43.093 | <0.001 | -0.1645 | 0.0438 | -3.7554 | <0.001 |
| Reaction time* | -0.0316 | 4.00E-04 | -77.9195 | <0.001 | -0.039 | 0.0093 | -4.1844 | <0.001 |
| Prospective memory | 0.2943 | 0.0094 | 31.1974 | <0.001 | 0.1516 | 0.1376 | 1.1018 | 0.2706 |
| Reasoning | 0.1677 | 0.008 | 20.8622 | <0.001 | 0.0505 | 0.144 | 0.351 | 0.7258 |
| Number memory | 0.1373 | 0.0094 | 14.5689 | <0.001 | 0.0373 | 0.2534 | 0.147 | 0.8834 |
| Notes: Coeff, Coefficient from Linear Mixed Model; N, number of participants; S.E. standard error   *Negative association as lower scores = better cognitive performance; **BOLD** indicates statistically significant  ^1^Adjusting waist circumference and history of vascular/heart conditions, along with age, education, gender, bodyweight and region | | | | | | | | |
